# Metabolic and Neural Mechanisms Underlying the Associations Between Gut *Bacteroides* and Cognition: A Large-Scale Functional Network Connectivity Study

**DOI:** 10.3389/fnins.2021.750704

**Published:** 2021-10-18

**Authors:** Shujun Zhang, Yinfeng Qian, Qian Li, Xiaotao Xu, Xueying Li, Chunli Wang, Huanhuan Cai, Jiajia Zhu, Yongqiang Yu

**Affiliations:** ^1^Department of Radiology, The First Affiliated Hospital of Anhui Medical University, Hefei, China; ^2^Research Center of Clinical Medical Imaging, Hefei, China; ^3^Anhui Provincial Institute of Translational Medicine, Hefei, China; ^4^Department of Clinical Laboratory, The First Affiliated Hospital of Anhui Medical University, Hefei, China

**Keywords:** gut *Bacteroides*, metabolic pathways, functional network connectivity, cognition, functional MRI

## Abstract

There is a proof-of-concept that microbial metabolites provide a molecular connection between the gut and the brain. Extensive research has established a link between gut *Bacteroides* and human cognition, yet the metabolic and neural mechanisms underlying this association remain largely unknown. Here, we collected fecal samples, resting-state functional MRI, and cognitive data from a large and homogeneous sample of 157 healthy young adults. 16S rRNA gene sequencing was conducted with abundances of *Bacteroides* and metabolic pathways quantified by species annotation and functional prediction analyses, respectively. Large-scale intra- and internetwork functional connectivity was measured using independent component analysis. Results showed that gut *Bacteroides* were related to multiple metabolic pathways, which in turn were associated with widespread functional network connectivity. Furthermore, functional network connectivity mediated the associations between some *Bacteroides-*related metabolic pathways and cognition. Remarkably, arginine and proline metabolism, phenylalanine metabolism, and biosynthesis of unsaturated fatty acids act as the key metabolic pathways that are most contributive, and the executive control and sensorimotor systems contribute most strongly at the neural level. Our findings suggest complex poly-pathway and poly-network processes linking *Bacteroides* to cognition, more generally yielding a novel conceptualization of targeting gut *Bacteroides* as an intervention strategy for individuals with cognitive impairment.

## Introduction

Over the last decade, some new insights on the mechanisms of microbiota-gut-brain (MGB) axis have been gained with the application multi-omics approaches. There is also extensive research on how specific bacteria contribute to the MGB axis. Among dominant beneficial bacteria, *Bacteroides* account for a major fraction of the gut bacteriome and play a prominent role in human health and disease (Wexler and Goodman, 2017; Zafar and Saier, 2021). They are critically implicated in the regulation of diverse metabolic processes, not only by metabolizing polysaccharides, oligosaccharides, volatile fatty acids, and short-chain fatty acids but also by influencing other microbes residing in the gut. As such, considerable effort has been directed toward investigating the possible metabolic mechanisms underlying the effects of gut *Bacteroides* on the central nervous system. For example, prior research suggests that specific types of lipopolysaccharides and endotoxins from *Bacteroides* impact the development of inflammatory neurodegeneration (Lukiw, 2016). Hartstra and colleagues showed that increases in fecal levels of *Bacteroides* were associated with an increase in brain dopamine transporter (Hartstra et al., 2020). Pilot studies have documented that gut *Bacteroides* affect glutamate metabolism, thereby further influencing cognitive function in dementia patients (Chang et al., 2020, 2021). Together, these studies provide a proof of concept that there are potential metabolic pathways through which gut *Bacteroides* exert their effects on the brain (Hofer, 2014).

A large number of clinical and preclinical studies have established that alterations in gut *Bacteroides* give rise to a wide range of neuropsychiatric disorders, such as attention-deficit/hyperactivity disorder (Wang L.J. et al., 2020), multiple sclerosis (Mirza et al., 2020), autism spectrum disorders (Hsiao et al., 2013), and Alzheimer’s disease (AD) (Lukiw, 2016). As a transdiagnostic signature of neuropsychiatric disorders (Williams and Sachdev, 2010; Sahakian et al., 2015; Gallagher et al., 2017), cognitive dysfunction has also been proved to be associated with dysregulation of gut *Bacteroides* (Saji et al., 2019; Luo et al., 2020; Liu et al., 2021). Of note, gut *Bacteroides* and dysregulation of their associated metabolism activities may be related to the pathogenesis of neurodevelopmental disorders (Dan et al., 2020; Jena et al., 2020; Tamana et al., 2021). Furthermore, *Bacteroides*-dominant gut microbiome can influence cognition by getting involved in the neurodevelopment during infancy (Carlson et al., 2018; Tamana et al., 2021). Therefore, illuminating the precise metabolic and neural mechanisms underlying the relation between gut *Bacteroides* and cognition is of high clinical and translational relevance.

Taking advantage of advanced neuroimaging techniques, there have been recent attempts to unpack the relationship between gut *Bacteroides* and brain structure and function. Saji et al. reported that patients with mild cognitive impairment had a higher prevalence of gut *Bacteroides* that was associated with more white matter hyperintensity and cortical and hippocampal atrophy (Saji et al., 2019). Contrasting with this finding, another study found that *Bacteroides* were associated with increased gray matter in the cerebellum, hippocampus, and frontal regions in healthy women (Tillisch et al., 2017). A resting-state functional magnetic resonance imaging (fMRI) study demonstrated that relative abundance of *Bacteroides* was negatively correlated with regional spontaneous neural activity of the cerebellum in patients with amnestic mild cognitive impairment (Liu et al., 2021). By leveraging seed-based functional connectivity approaches, researchers have revealed associations of gut *Bacteroides* with reduced connectivity between core reward regions in patients with obesity after laparoscopic sleeve gastrectomy (Dong et al., 2020) and lower connectivity between the dorsolateral prefrontal cortex and anterior medial frontal cortex in patients with major depressive disorder (Strandwitz et al., 2019). While our previous work indicates significant effects of microbial diversity and enterotypes on functional connectivity between and within large-scale neural networks (Cai et al., 2021), the exact relationship between gut *Bacteroides* and large-scale functional network connectivity has yet to be determined.

**FIGURE 1:**
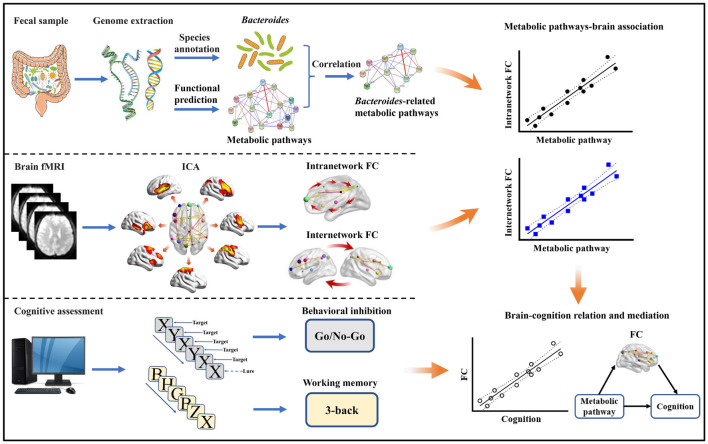
Flowchart of the study design. FC, functional connectivity; fMRI, functional magnetic resonance imaging; ICA, independent component analysis.

Motivated by the proof-of-concept that microbial metabolites provide a molecular connection between the gut and the brain (Hofer, 2014), we collected fecal samples, resting-state fMRI, and cognitive data from a large and homogeneous sample of healthy young adults. 16S rRNA gene sequencing was conducted with abundances of *Bacteroides* and metabolic pathways quantified by species annotation and functional prediction analyses, respectively. Large-scale intra- and internetwork functional connectivity was measured using independent component analysis (ICA) (Wang C. et al., 2020), as converging evidence has emphasized the pivotal role of functional network connectivity in cognition (Park and Friston, 2013). By a combined analysis of these data, the objectives of this investigation were four-fold. First, we set out to find the metabolic pathways strongly associated with gut *Bacteroides*. Second, we aimed to assess the associations of *Bacteroides*-related metabolic pathways with functional network connectivity. Third, we sought to investigate the potential associations between metabolic pathways-linked functional connectivity and cognition. Finally, we attempted to determine the mediative role of these identified functional connectivity markers in accounting for the associations between *Bacteroides*-related metabolic pathways and cognition. A systematic flowchart of the study design is illustrated in Figure 1. Here, we hypothesized that gut *Bacteroides* would have consequences on cognition via key metabolic pathways and widespread functional network connectivity.

## Materials and Methods

### Participants

A total of 157 healthy young adults were recruited by advertisement. All participants met the inclusion criteria of Chinese Han, right handedness, and within a restricted age range of 18–30 years. Exclusion criteria included neuropsychiatric or severe somatic disorder, a history of alcohol or drug abuse, regular smoker, current medication (e.g., antibiotics or sedative hypnotics) within a month, pregnancy, MRI contraindications, and a family history of psychiatric illness among first-degree relatives. The MINI-International Neuropsychiatric Interview (M.I.N.I.) and Alcohol Use Disorders Identification Test (AUDIT) were used in the process of excluding participants. This study was approved by the ethics committee of The First Affiliated Hospital of Anhui Medical University. Written informed consent was obtained from all participants after they had been given a complete description of the study. Detailed data of the participants are listed in Table 1.

**TABLE 1:** Demographic, behavioral, and gut microbial characteristics of the sample.

Characteristics	Mean ± SD	Range
Gender (male/female)	80/77	–
Age (years)	22.3 ± 2.4	18–28
Education (years)	15.8 ± 1.9	12–20
BMI (kg/m^2^)	21.44 ± 3.20	15.42–36.99
FD (mm)	0.12 ± 0.05	0.04–0.40
Relative abundance of *Bacteroides*	0.22 ± 0.20	0.0004–0.6581
3-back task performance		
Accuracy	0.72 ± 0.16	0.15–0.98
Reaction time (ms)	768.9 ± 175.2	230.2–1179.9
Go/No-Go task performance		
RT_Go (ms)	432.83 ± 69.57	256.73–591.64
Acc_No-Go	0.59 ± 0.19	0.05–1.00

*The data are presented as the mean ± standard deviation. Acc_No-Go, accuracy in “No-Go” conditions; BMI, body mass index; FD, frame-wise displacement; RT_Go, mean reaction time of correct responses in “Go” conditions.*

### Working Memory Assessment

The letter 3-back task was conducted on a computer to assess working memory using E-Prime 2.0^1^ (Owen et al., 2005). During the task, each participant viewed a series of letters that were presented sequentially, and the presentation time of each letter stimulus was 200 ms with an interstimulus interval of 1,800 ms. Participants were instructed to press a button on the right with their middle fingers if the letter that appeared on the screen was identical to the one presented 3 letters earlier and otherwise to press a button on the left with their index fingers. The task consisted of 60 trials. Before the formal test, participants were verbally instructed and had a practice test to ensure that they understood the task. The accuracy and mean reaction time of correct responses were used as the indices of working memory performance.

### Behavioral Inhibition Evaluation

The Go/No-Go task was conducted on a computer to assess the ability of behavioral inhibition using E-Prime 2.0 (see text footnote 1) (Kaufman et al., 2003). During the task, the letter X or Y was presented at a frequency of 1 Hz on the screen. In “Go” conditions, the current letter is different from the previous one and participants should respond quickly by pressing the button within 900 ms. In “No-Go” conditions (10% of all trials), the current letter is the same as the previous one and participants cannot press the button; if one presses the button, it would be counted as an error. The Go/No-Go task consisted of a practice test and a formal test. There were 20 trials (15 “Go” trials and 5 “No-Go” trials) in the practice test. If a participant responds correctly in 3 “No-Go” trials, he or she can shift to the formal test; otherwise, the participant needs to restart the practice test. The formal test was divided into two groups with 210 trials in each group and 30 s break between the two groups. It took about 12 min for the Go/No-Go task. The accuracy in “No-Go” conditions (Acc_No-Go) as well as the mean reaction time of correct responses in “Go” conditions (RT_Go) were used as the indices of task performance.

### Magnetic Resonance Imaging Data Acquisition and Preprocessing

Resting-state BOLD fMRI and high-resolution structural MRI data were obtained using a 3.0-Tesla MR system (Discovery MR750w, General Electric, Milwaukee, WI, United States) within 1 or 2 days after cognition assessment. Resting-state BOLD data were preprocessed using SPM12 and Data Processing & Analysis for Brain Imaging (DPABI)^2^. The details are described in the Supplementary Methods.

### Independent Component Analysis

ICA was employed to parcellate the preprocessed fMRI data with the GIFT toolbox,^3^ and the number of independent components (*N* = 26) was estimated automatically by the software using the minimum description length criteria. Spatial ICA decomposes the participant data into linear mixtures of spatially independent components that exhibit a unique time course profile. This was achieved by using two data reduction steps. First, principal component analysis was applied to reduce the subject-specific data into 39 principal components. Next, reduced data of all subjects were concatenated across time and decomposed into 26 independent components using the infomax algorithm. To ensure estimation stability, the infomax algorithm was repeated 20 times in ICASSO,^4^ and the most central run was selected and analyzed further. Finally, participant-specific spatial maps and time courses were obtained using the GICA back reconstruction approach.

We identified as functional networks several independent components that had peak activations in gray matter; showed low spatial overlap with known vascular, ventricular, motion, and susceptibility artifacts; and exhibited primarily low-frequency power. This selection procedure yielded 14 functional networks out of the 26 independent components obtained (Supplementary Figure 1): anterior and posterior default mode networks (aDMN and pDMN); executive control network (ECN); left and right frontoparietal networks (lFPN and rFPN); salience network (SN); dorsal and ventral attention networks (DAN and VAN); dorsal and ventral sensorimotor networks (dSMN and vSMN); auditory network (AN); and medial, lateral, and posterior visual networks (mVN, lVN, and pVN).

Before internetwork functional connectivity calculation, the following additional postprocessing steps were performed on the time courses of selected functional networks: (1) detrending linear, quadratic, and cubic trends; (2) despiking detected outliers; and (3) low-pass filtering with a cut-off frequency of 0.15 Hz. Then, internetwork functional connectivity was estimated as the Pearson correlation coefficients between pairs of time courses of the functional networks, resulting in a symmetric 14 × 14 correlation matrix for each subject. Finally, correlations were transformed to *Z*-scores using Fisher’s transformation to improve the normality. Intranetwork connectivity was examined via the spatial maps, indexing the contribution of the time course to each voxel comprising a given component.

### Fecal Samples Collection and Gut Microbiota Analysis

Fecal samples were collected in sterilized tubes and stored immediately in a −80°C freezer within 1 day before or after MRI examination. Microbial genome DNA was extracted from the fecal samples using a QIAamp DNA Stool Mini Kit (Qiagen Inc., Hilden, Germany). To construct the Polymerase Chain Reaction (PCR)-based 16S rRNA amplicon library for sequencing, PCR enrichment of the V4 hypervariable region of 16S rRNA gene was performed with the forward primer 515F (5′-GTGCCAGCMGCCGCGGTAA-3′) and reverse primer 806R (5′-GGACTACHVGGGTWTCTAAT-3′). The qualified amplicon mixture was then sequenced on the MiSeq platform with the PE250 sequencing strategy. Before the 16S rRNA data analysis, raw reads were filtered to remove adaptors and low-quality and ambiguous bases, and then paired-end reads were added to tags by the Fast Length Adjustment of Short reads program (FLASH, v1.2.11) (Magoc and Salzberg, 2011). The tags were clustered into operational taxonomic units (OTUs) with a cutoff value of 97% using UPARSE software (v9.1.13) (Edgar, 2013) and chimera sequences were compared with the Gold database using UCHIME (v4.2.40) (Edgar et al., 2011) to detect. Then, the representative sequence from each OTU cluster was obtained. These OTU representative sequences were taxonomically classified using Ribosomal Database Project (RDP) Classifier (v.2.2) (Wang et al., 2007) with a minimum confidence threshold of 0.8, and the training database was the Greengene Database (v201305) (DeSantis et al., 2006). The USEARCH_global (Edgar, 2010) was used to compare all tags back to OTU to get the OTU abundance statistics table of each sample. Microbial relative abundances were then quantified at the genus level and we focused our analysis on the *Bacteroides*. PICRUSt2 (phylogenetic investigation of communities by reconstruction of unobserved states, version 2.30) was performed to predict functional composition of microbial communities from 16S data (Langille et al., 2013; Douglas et al., 2019). PICRUSt2 predictions were based on a database of gene families and reference genomes, i.e., Kyoto Encyclopedia of Genes and Genomes (KEGG) orthology (KO)^5^ (Kanehisa et al., 2012). Predicted KEGG pathways represent the functional profiling of microbial communities. Based on a review of the literature (Rhee et al., 2009; Dinan and Cryan, 2017; Martin et al., 2018; Cryan et al., 2019), 33 brain-related metabolic pathways (Supplementary Table 1) were selected and relative abundances of these metabolic pathways were estimated for subsequent analysis.

### Statistical Analysis

The statistical descriptive analyses of demographic and behavioral data were conducted using the SPSS 23.0 software package (SPSS, Chicago, IL, United States). A multi-stage approach was adopted to analyze the data of *Bacteroides*, metabolic pathways, neuroimaging (intra- and internetwork functional connectivity), and cognitive functions (working memory and behavioral inhibition). First, we tested for the associations between *Bacteroides* and brain-related metabolic pathways using partial correlation analyses with age and sex as nuisance covariates. For metabolic pathways associated with *Bacteroides*, we further examined their associations with functional connectivity using partial correlations adjusting for age, sex and frame-wise displacement (FD). For internetwork functional analysis, multiple comparisons were corrected by false discovery rate (FDR) with a corrected significance level of *p* < 0.05. For intranetwork functional analysis, all participants’ spatial maps for each functional network were initially entered into a random-effect one-sample *t*-test. Brain regions were considered to be within each network if they met a height threshold of *p* < 0.05 corrected for multiple comparisons using a family-wise error (FWE) method. Next, we performed the above-described correlation analyses in a voxel-wise manner within each network. Multiple comparisons were corrected using the cluster-level FWE method, resulting in a cluster defining threshold of *p* = 0.001 and a corrected cluster significance of *p* < 0.05. Second, for inter- and intranetwork functional connectivity showing correlations with the *Bacteroides*-related metabolic pathways, we further examined their associations with cognitive functions using partial correlations adjusting for age, sex, FD, and educational level. Finally, to further test whether the relationship between the *Bacteroides*-related metabolic pathways and cognitive functions was mediated by functional connectivity, mediation analysis was performed using the PROCESS macro^6^ (Hayes, 2009). In the mediation models, all paths were reported as unstandardized ordinary least squares regression coefficients, namely, total effect of X on Y (c) = indirect effect of X on Y through M (a × b) + direct effect of X on Y (c’). The significance analysis was based on 10,000 bootstrap realizations and a significant indirect effect is indicated when the bootstrap 95% confidence interval (CI) does not include zero. In the mediation analysis, only variables that showed a significant correlation with others were considered independent (*Bacteroides*-related metabolic pathways), dependent (cognitive functions), or mediating (intra- and internetwork functional connectivity) variables. Age, sex, FD, and educational level were considered nuisance variables.

## Results

### Associations Between Gut *Bacteroides* and Brain-Related Metabolic Pathways

**FIGURE 2:**
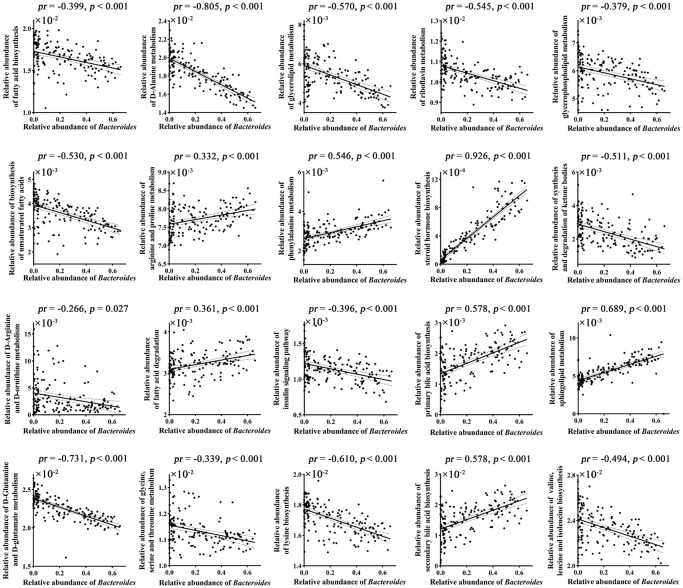
Correlations between gut *Bacteroides* and brain-related metabolic pathways. *p* values were corrected for multiple comparisons using the Bonferroni method. *pr*, partial correlation coefficient.

Correlation analyses revealed that 20 out of 33 brain-related metabolic pathways were significantly correlated with *Bacteroides* (*p* < 0.05, Bonferroni corrected; Figure 2 and Supplementary Table 1).

### Associations Between *Bacteroides*-Related Metabolic Pathways and Intranetwork Functional Connectivity

**FIGURE 3:**
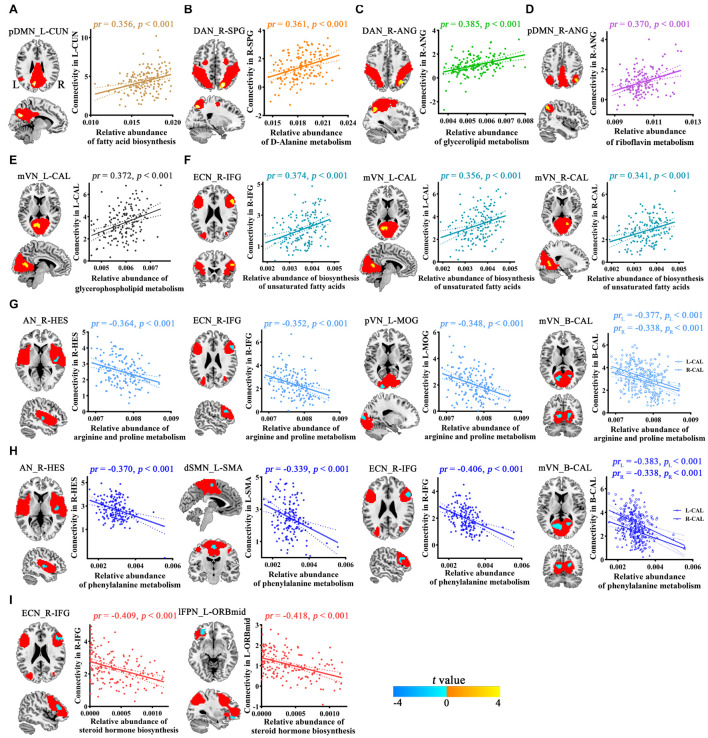
Correlations between *Bacteroides*-related metabolic pathways and intranetwork functional connectivity. Red masks represent spatial maps of corresponding functional networks. AN, auditory network; ANG, angular gyrus; B, bilateral; CAL, calcarine fissure and surrounding cortex; CUN, cuneus; DAN, dorsal attention network; dSMN, dorsal sensorimotor network; ECN, executive control network; HES, Heschl’s gyrus; IFG, inferior frontal gyrus; L, left; lFPN, left frontoparietal network; MOG, middle occipital gyrus; mVN, medial visual network; ORBmid, orbital part of middle frontal gyrus; pDMN, posterior default mode network; pVN, posterior visual network; R, right; SMA, supplementary motor area; SPG, superior parietal gyrus.

Voxel-wise intranetwork functional connectivity analyses demonstrated that 9 from 20 *Bacteroides*-related metabolic pathways were significantly correlated with connectivity within multiple functional networks (*p* < 0.05, cluster-level FWE corrected; Figure 3 and Table 2). Specifically, fatty acid biosynthesis was positively correlated with pDMN (Figure 3A), D-Alanine metabolism with DAN (Figure 3B), glycerolipid metabolism with DAN (Figure 3C), riboflavin metabolism with pDMN (Figure 3D), glycerophospholipid metabolism with mVN (Figure 3E), and biosynthesis of unsaturated fatty acids with ECN and mVN (Figure 3F). Arginine and proline metabolism was negatively correlated with AN, ECN, pVN and mVN (Figure 3G), phenylalanine metabolism with AN, dSMN, ECN and mVN (Figure 3H), and steroid hormone biosynthesis with ECN and lFPN (Figure 3I).

**TABLE 2:** Brain regions showing significant correlations between *Bacteroides*-related metabolic pathways and intranetwork functional connectivity.

*Bacteroides*-related metabolic pathways	Functional network	Brain region	Cluster size (voxels)	Peak *t*-values	Coordinates in MNI (x, y, z)
Fatty acid biosynthesis	pDMN	L-CUN	32	4.32	−9, −72, 30
D-Alanine metabolism	DAN	R-SPG	38	4.75	21, −66, 48
Glycerolipid metabolism	DAN	R-ANG	44	4.41	36, −60, 42
Riboflavin metabolism	pDMN	R-ANG	26	4.62	42, −60, 48
Glycerophos pholipid metabolism	mVN	L-CAL	84	4.90	−3, −63, 12
Biosynthesis of unsaturated fatty acids	mVN	L-CAL	69	4.38	−3, −63, 12
	mVN	R-CAL	46	3.83	15, −57, 0
	ECN	R-IFG	35	4.01	54, 21, 24
Arginine and proline metabolism	AN	R-HES	37	−4.39	42, −15, 6
	ECN	R-IFG	32	−4.10	57, 21, 27
	pVN	L-MOG	36	−4.81	−18, −96, 9
	mVN	L-CAL	112	−4.68	−15, −66, 9
	mVN	R-CAL	47	−3.85	21, −63, 12
Phenylalanine metabolism	AN	R-HES	41	−4.66	42, −15, 9
	dSMN	L-SMA	29	−4.42	0, −12, 60
	ECN	R-IFG	51	−4.33	54, 24, 24
	mVN	L-CAL	120	−4.78	−12, −63, 9
	mVN	R-CAL	34	−4.32	21, −60, 15
Steroid hormone biosynthesis	ECN	R-IFG	56	−4.16	45, 33, 18
	lFPN	L-ORBmid	38	−5.14	−30, 42, −12

*AN, auditory network; ANG, angular gyrus; B, bilateral; CAL, calcarine fissure and surrounding cortex; CUN, cuneus; dSMN, dorsal sensorimotor network; DAN, dorsal attention network; ECN, executive control network; HES, Heschl’s gyrus; IFG, inferior frontal gyrus; L, left; lFPN, left frontoparietal network; MOG, middle occipital gyrus; mVN, medial visual network; ORBmid, orbital part of middle frontal gyrus; pDMN, posterior default mode network; pVN, posterior visual network; R, right; SMA, supplementary motor area; SPG, superior parietal gyrus.*

### Associations Between *Bacteroides*-Related Metabolic Pathways and Internetwork Functional Connectivity

**FIGURE 4:**
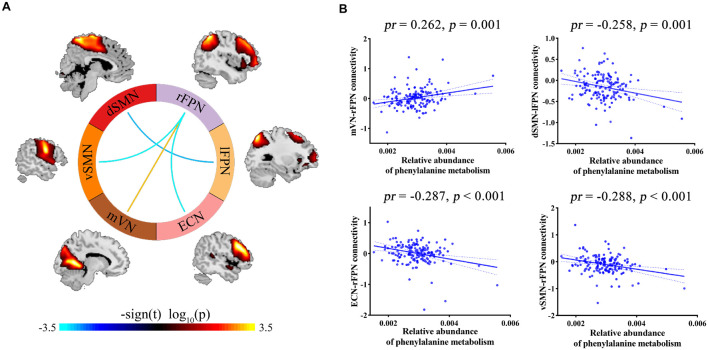
**(A)** Internetwork functional connectivity correlating with phenylalanine metabolism. **(B)** Scatter plots showing the correlations between phenylalanine metabolism and internetwork functional connectivity. dSMN, dorsal sensorimotor network; ECN, executive control network; lFPN, left frontoparietal network; mVN, medial visual network; *pr*, partial coefficient; rFPN, right frontoparietal network; vSMN, ventral sensorimotor network.

Correlation analyses revealed significant associations between phenylalanine metabolism and internetwork functional connectivity (*p* < 0.05, FDR corrected; Figure 4). Specifically, phenylalanine metabolism was positively correlated with functional connectivity between mVN and rFPN, as well as negatively correlated with connectivity between dSMN and lFPN, between ECN and rFPN, and between vSMN and rFPN. However, there were no significant correlations between other *Bacteroides*-related metabolic pathways and internetwork functional connectivity.

### The Mediative Role of Functional Connectivity in Accounting for Associations Between *Bacteroides*-Related Metabolic Pathways and Cognition

**FIGURE 5:**
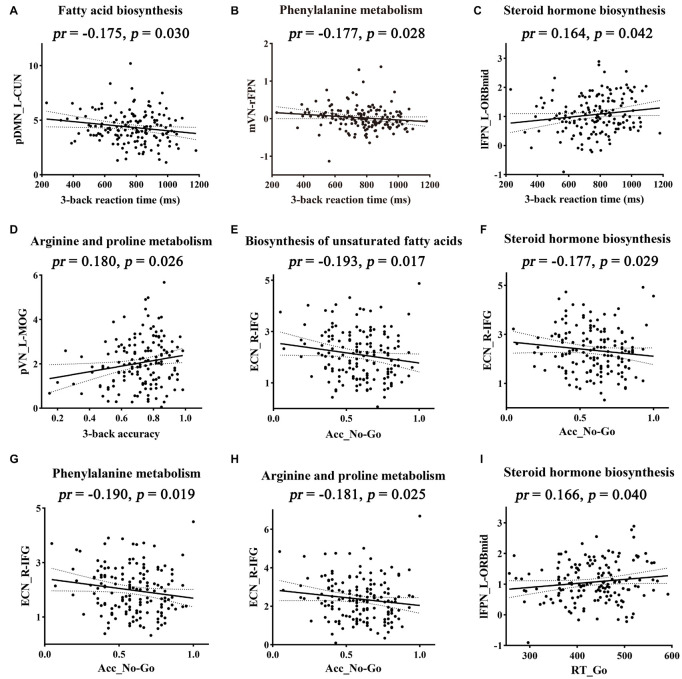
Correlations between functional connectivity and cognition. Metabolic pathways above the scatter plots were associated with the corresponding functional connectivity. Acc_No-Go, the accuracy in “No-Go” conditions; CUN, cuneus; ECN, executive control network; IFG, inferior frontal gyrus; L, left; lFPN, left frontoparietal network; MOG, middle occipital gyrus; mVN, medial visual network; ORBmid, orbital part of middle frontal gyrus; pDMN, posterior default mode network; *pr*, partial correlation coefficient; pVN, posterior visual network; R, right; rFPN, right frontoparietal network; RT_Go, the mean reaction time of correct responses in “Go” conditions.

**FIGURE 6:**
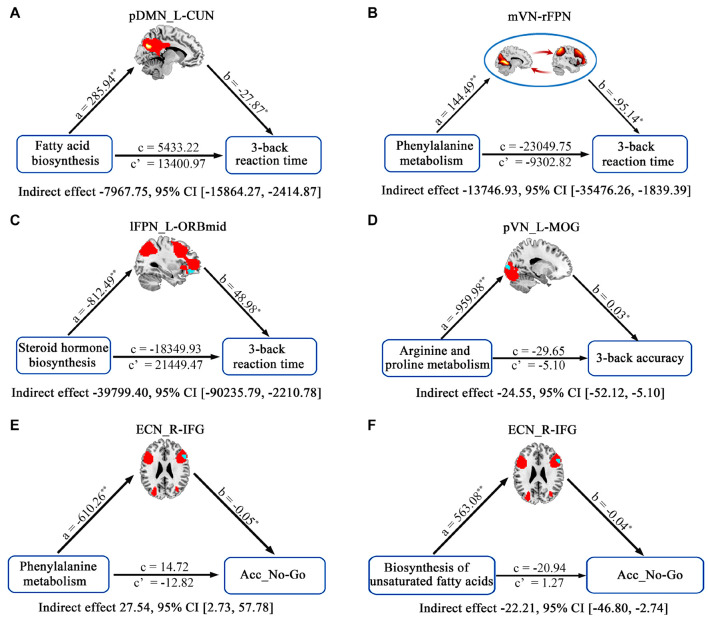
The mediative role of functional connectivity in accounting for associations between *Bacteroides*-related metabolic pathways and cognition. In the mediation models, all paths were reported as unstandardized ordinary least squares regression coefficients, namely, total effect of X on Y (c) = indirect effect of X on Y through M (a × b) + direct effect of X on Y (c’). *p < 0.05 and **p < 0.01. Acc_No-Go, the accuracy in “No-Go” conditions; CI, confidence interval; CUN, cuneus; ECN, executive control network; IFG, inferior frontal gyrus; L, left; lFPN, left frontoparietal network; MOG, middle occipital gyrus; mVN, medial visual network; ORBmid, orbital part of middle frontal gyrus; pDMN, posterior default mode network; *pr*, partial correlation coefficient; pVN, posterior visual network; R, right; rFPN, right frontoparietal network.

The relationships between cognition and functional connectivity related to *Bacteroides*-related metabolic pathways were further investigated. With regard to working memory, 3-back reaction time was found to be negatively correlated with functional connectivity within pDMN related to fatty acid biosynthesis (Figure 5A) and mVN-rFPN connectivity related to phenylalanine metabolism (Figure 5B), and positively correlated with connectivity within lFPN related to steroid hormone biosynthesis (Figure 5C). 3-back accuracy showed a significant positive correlation with connectivity within pVN related to arginine and proline metabolism (Figure 5D). Further mediation analyses revealed that functional connectivity (intra-pDMN, mVN-rFPN, and intra-lFPN) mediated the relationships between *Bacteroides*-related metabolic pathways (fatty acid biosynthesis, phenylalanine metabolism, and steroid hormone biosynthesis) and 3-back reaction time (Figures 6A–C). Intra-pVN connectivity mediated the relationship between arginine and proline metabolism and 3-back accuracy (Figure 6D).

With respect to behavioral inhibition, Acc_No-Go was found to be negatively correlated with functional connectivity within ECN related to biosynthesis of unsaturated fatty acids, steroid hormone biosynthesis, phenylalanine metabolism, and arginine and proline metabolism (Figures 5E–H). In addition, RT_Go showed a significant positive correlation with connectivity within lFPN related to steroid hormone biosynthesis (Figure 5I). Further mediation analyses revealed that intra-ECN connectivity mediated the relationships between *Bacteroides*-related metabolic pathways (phenylalanine metabolism and biosynthesis of unsaturated fatty acids) and Acc_No-Go (Figures 6E,F).

### Correlation Network of Gut *Bacteroides*, Metabolic Pathways, Functional Connectivity, and Cognition

**FIGURE 7:**
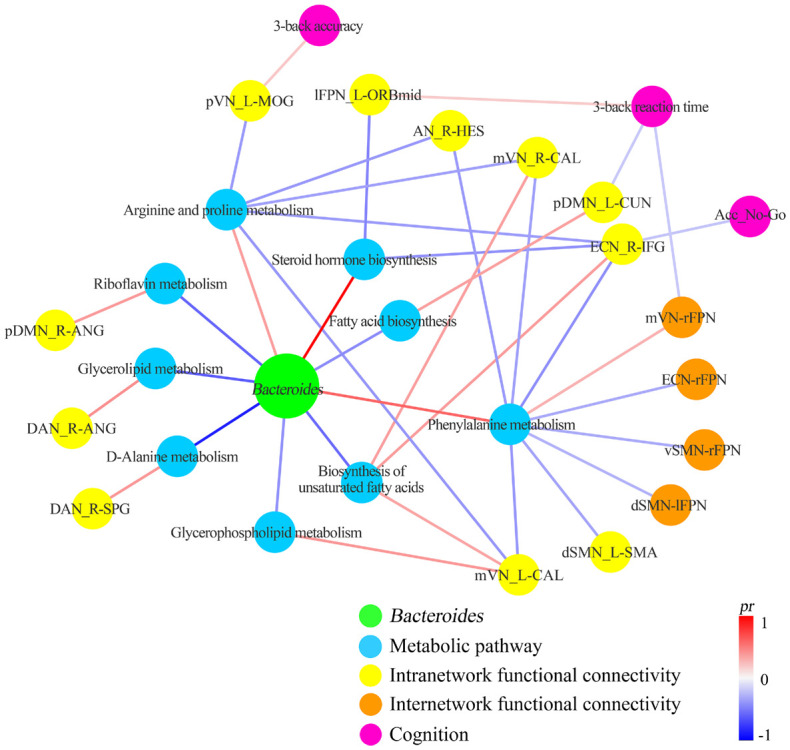
Correlation network of gut *Bacteroides*, metabolic pathways, functional connectivity, and cognition. Acc_No-Go, the accuracy in “No-Go” conditions; AN, auditory network; ANG, angular gyrus; B, bilateral; CAL, calcarine fissure and surrounding cortex; CUN, cuneus; DAN, dorsal attention network; dSMN, dorsal sensorimotor network; ECN, executive control network; HES, Heschl’s gyrus; IFG, inferior frontal gyrus; L, left; lFPN, left frontoparietal network; MOG, middle occipital gyrus; mVN, medial visual network; ORBmid, orbital part of middle frontal gyrus; pDMN, posterior default mode network; *pr*, partial coefficient; pVN, posterior visual network; R, right; SMA, supplementary motor area; SPG, superior parietal gyrus.

A correlation network of gut *Bacteroides*, metabolic pathways, functional connectivity, and cognition is summarized in Figure 7. Visual inspection indicates that arginine and proline metabolism, phenylalanine metabolism, and biosynthesis of unsaturated fatty acids act as the key metabolic pathways linking gut *Bacteroides* to multiple functional connectivity within and between large-scale networks, which in turn have an impact on cognition. At the neural level, the executive control (ECN, lFPN, and rFPN) and sensorimotor (dSMN, vSMN, AN, mVN and pVN) systems are preferentially affected by multiple *Bacteroides*-related metabolic pathways.

## Discussion

This study is the first to investigate the underlying metabolic and neural mechanisms by which gut *Bacteroides* interact with cognition. Our findings of the involvement of multiple metabolic pathways and widespread functional network connectivity highlight the complex poly-pathway and poly-network processes linking *Bacteroides* to cognition. Regarding the metabolic pathways, arginine and proline metabolism, phenylalanine metabolism, and biosynthesis of unsaturated fatty acids contribute most to the associations; the executive control and sensorimotor systems are considered as being most contributive at the neural level. The present study, extending our previous work (Cai et al., 2021) in an elegant way, may shed light on the gut *Bacteroides*-metabolic pathways-functional connectivity-cognition relationships.

There have been three resting-state fMRI precedents attempting to uncover the relationship between gut *Bacteroides* and brain functional connectivity (Curtis et al., 2019; Strandwitz et al., 2019; Dong et al., 2020). In those earlier studies, however, functional connectivity was measured using a classical hypothesis-driven seed-based approach, which is limited in its ability to capture functional connectivity patterns globally since the seed regions must be selected *ad hoc*. In this research, we preferred the data-driven ICA method over the seed-based analysis because the former automatically separates the signals of the whole brain into statistically independent components, which facilitates a more thorough characterization of the whole-brain functional connectome (van de Ven et al., 2004; Damoiseaux et al., 2006). Moreover, our findings of poly-network involvement corroborate the utility of functional network connectivity as an unbiased and reliable biomarker for exploring the gut microbiota-brain relationship.

Understanding microbiota function may be more direct and important for elucidating the effects of microbial taxa on downstream brain organization and host behavior (Nguyen et al., 2021). Among functional pathways affected by *Bacteroides*, phenylalanine metabolism, biosynthesis of unsaturated fatty acids, and arginine and proline metabolism serve as the key metabolic pathways with relation to multiple functional connectivity within and between large-scale networks, which in turn have an impact on cognition. Phenylalanine metabolism has a critical role in brain development and subserves the maintenance of normal cognitive and behavioral performance. Phenylalanine metabolism disorder can cause hyperphenylalaninemia that leads to intellectual impairment and behavioral disturbance (Ashe et al., 2019). The brain is highly enriched with fatty acids, including the unsaturated fatty acids that are largely esterified to the phospholipid cell membrane. The unsaturated fatty acids participate in signal transduction, such that they can regulate several neural processes such as neurotransmission, cell survival and neuroinflammation, and thereby mood and cognition (Bazinet and Laye, 2014). Alterations of the unsaturated fatty acids are evident in various brain disorders, including Alzheimer’s disease and major depressive disorder. An animal study showed that early intake of long-chain polyunsaturated fatty acids could preserve brain functional connectivity and improve cognition in diet-induced obesity (Arnoldussen et al., 2016), which is partly consistent with our observation that biosynthesis of unsaturated fatty acids affected behavioral inhibition via regulating intra-ECN functional connectivity. Arginine is a precursor for the synthesis not only of proteins but also of nitric oxide, urea, polyamines, proline, glutamate, creatine and agmatine (Wu and Morris, 1998). Of note, it has been well documented that nitric oxide involves neurotransmission, synaptic plasticity and learning and memory (Susswein et al., 2004; Feil and Kleppisch, 2008; Zhou and Zhu, 2009), as well as represents a major regulator of cerebral blood flow (Hariharan et al., 2019). Concurrently, evidence from animal studies suggests neurochemical and behavioral effects of proline metabolism (Wyse and Netto, 2011). These previous efforts, taken with our data, may endorse the importance of arginine and proline metabolism in regulating brain function and human behavior. Complementing and extending the prior literature, the present findings establish an inherent link between gut *Bacteroides* and the three metabolic pathways, supporting the notion that microbial metabolites might bridge the gap between the gut and the brain. Other *Bacteroides*-related metabolic pathways have also been shown to affect brain structure and/or function in direct or indirect ways, such as steroid hormone biosynthesis (Jaggar et al., 2020; Park et al., 2021), fatty acid biosynthesis (Edmond et al., 1998; Schwiertz et al., 2010; Singh, 2019), glycerolipid metabolism (Assmann et al., 2020), glycerophospholipid metabolism (Brown et al., 2019; Zheng et al., 2020), riboflavin metabolism (Spector, 1980; Radjabzadeh et al., 2020), and D-Alanine metabolism (Popiolek et al., 2018; Quagliariello et al., 2018). Specifically, glycerolipid and glycerophospholipid metabolisms determine the extent of axon regeneration, since injured neurons require a large supply of lipids for membrane formation (Yang et al., 2020). In addition, Zheng and colleagues indicated that gut microbiome may participate in the onset of depressive-like behaviors by modulating peripheral and central glycerophospholipid metabolism (Zheng et al., 2020). More importantly, evidence suggests that gut *Bacteroidetes* are the only gut commensal known to produce sphingolipids (Olsen and Jantzen, 2001; Brown et al., 2019). This is in line with our finding that gut *Bacteroides* were correlated with glycerolipid and glycerophospholipid metabolisms. Thus, one may speculate that gut *Bacteroides* might play a critical role in the axon development and regeneration by influencing glycerolipid and glycerophospholipid metabolisms, serving as a potential neural mechanism via which gut *Bacteroides* affect brain structure and function. Collectively, the results of this study are an essential step toward the identification of microbial and metabolic predictors of brain function and cognition, as well as determining the potential of targeting gut *Bacteroides* as a therapeutic strategy in patients with cognitive decline.

We demonstrate that multiple *Bacteroides*-related metabolic pathways preferentially influence functional connectivity of the executive control and sensorimotor systems. The executive control system mainly consists of the prefrontal and lateral parietal cortices, which are thought to be involved in a variety of goal-oriented cognitive-control processes such as working memory, inhibitory control, and attention (Song et al., 2013; Cai et al., 2021). In agreement with the concept, we found that functional connectivity of the executive control system mediated the relation of *Bacteroides*-related metabolic pathways with working memory and behavioral inhibition. This finding may add to the current knowledge by identifying the fundamental brain connectivity substrates underlying microbial metabolism-cognition associations. Meanwhile, we observed that functional connectivity of the sensorimotor system, and VN in particular, also contributed to the associations. On one hand, it is often assumed that specific cognitive processes arise from efficient integration and segregation of different functional networks. On the other hand, the prominent VN involvement may be due to the fact that detecting and processing of visual stimuli are a prerequisite for the 3-back and Go/No-Go tasks.

There are several limitations that should be mentioned. First, the cross-sectional design limits our ability to make causal inferences. Future prospective longitudinal studies are needed to resolve causality of the complex gut microbiota-metabolic pathways-brain-cognition relationship. Second, our study sample was selected from a group of educated young adults, thus limiting the generalizability of the findings. Third, microbial function was predicted based on the 16S data, which does not provide direct information about the functional potential of gut microbiota. Future studies should incorporate shotgun metagenomic sequencing, which directly and accurately measures microbial genetic functional potential, to validate our preliminary results. Finally, we did not collect data of fecal or circulating metabolites to further verify the identified *Bacteroides*-related metabolic pathways, which await confirmation using metabolomics.

In summary, our data reveal multiple metabolic pathways and widespread functional network connectivity that are informative about the underlying metabolic and neural mechanisms through which gut *Bacteroides* influence cognition. These findings suggest complex poly-pathway and poly-network processes linking *Bacteroides* to cognition, more generally yielding a novel conceptualization of targeting gut *Bacteroides* as an intervention strategy for individuals with cognitive impairment.

## Data Availability Statement

The raw data supporting the conclusions of this article will be made available by the authors, without undue reservation.

## Ethics Statement

The studies involving human participants were reviewed and approved by the Ethics Committee of The First Affiliated Hospital of Anhui Medical University. The patients/participants provided their written informed consent to participate in this study.

## Author Contributions

SZ, YQ, JZ, and YY conceptualized and designed the study. SZ was responsible for conducting the analyses, preparing the first draft of the manuscript, and preparing the manuscript for submission. JZ and YY were responsible for obtaining funding for the study, supervising the analyses, and editing drafts of the manuscript. SZ, YQ, QL, XX, XL, CW, and HC were responsible for data collection and initial data preprocessing. All authors contributed to and approved the final manuscript.

## Conflict of Interest

The authors declare that the research was conducted in the absence of any commercial or financial relationships that could be construed as a potential conflict of interest.

## Publisher’s Note

All claims expressed in this article are solely those of the authors and do not necessarily represent those of their affiliated organizations, or those of the publisher, the editors and the reviewers. Any product that may be evaluated in this article, or claim that may be made by its manufacturer, is not guaranteed or endorsed by the publisher.

## Footnotes

1
http://www.pstnet.com/eprime.cfm

2
http://rfmri.org/dpabi

3
http://mialab.mrn.org/software/gift/

4
http://research.ics.tkk.fi/ica/icasso/

5
http://www.genome.jp/kegg/

6
http://www.processmacro.org/
