# Supplementary material for: Metabolic and Neural Mechanisms Underlying the Associations Between Gut Bacteroides and Cognition: A Large-Scale Functional Network Connectivity Study
Source: Front Neurosci. 2021 Oct 18;15:750704. doi: 10.3389/fnins.2021.750704 (PMC8558260; doi:10.3389/fnins.2021.750704)
Supplement: Supplementary file 1 [file Data_Sheet_1.doc]

**Supplementary Materials**

**Supplementary methods**

**MRI data acquisition**

MRI scans were obtained using a 3.0-Tesla MR system (Discovery MR750w, General Electric, Milwaukee, WI, USA) with a 24-channel head coil within 1 or 2 days after cognition assessment. Earplugs were used to reduce scanner noise, and tight but comfortable foam padding was used to minimize head motion. High-resolution 3D T1-weighted structural images were acquired by employing a brain volume (BRAVO) sequence with the following parameters: repetition time (TR) = 8.5 ms; echo time (TE) = 3.2 ms; inversion time (TI) = 450 ms; flip angle = 12°; field of view (FOV) = 256 mm × 256 mm; matrix size = 256 × 256; slice thickness = 1 mm, no gap; 188 sagittal slices. Resting-state blood-oxygen-level-dependent (BOLD) fMRI data were acquired using a gradient-echo single-shot echo planar imaging (GRE-SS-EPI) sequence with the following parameters: TR = 2000 ms; TE = 30 ms; flip angle = 90°; FOV = 220 mm × 220 mm; matrix size = 64 × 64; slice thickness = 3 mm, slice gap = 1 mm; 35 interleaved axial slices; 185 volumes. All images were visually inspected to ensure that only images without visible artifacts were included in subsequent analyses.

**fMRI data preprocessing**

Resting-state BOLD data were preprocessed using Statistical Parametric Mapping software (SPM12, [http://www.fil.ion.ucl.ac.uk/spm](http://www.fil.ion.ucl.ac.uk/spm/software/spm8/)) and Data Processing & Analysis for Brain Imaging (DPABI, [http://rfmri.org/dpabi](http://rfmri.org/DPARSF)) (Yan et al. 2016). The first 10 volumes for each participant were discarded to allow the signal to reach equilibrium and the participants to adapt to the scanning noise. The remaining volumes were corrected for the acquisition time delay between slices. Then, realignment was performed to correct the motion between time points. Head motion parameters were computed by estimating the translation in each direction and the angular rotation on each axis for each volume. All participants’ BOLD data were within the defined motion thresholds (i.e., translational or rotational motion parameters less than 2 mm or 2°). We also calculated frame-wise displacement (FD), which indexes the volume-to-volume changes in head position. In the normalization step, individual structural images were firstly co-registered with the mean functional image; then the transformed structural images were segmented and normalized to the Montreal Neurological Institute (MNI) space using a high-level nonlinear warping algorithm, that is, the diffeomorphic anatomical registration through the exponentiated Lie algebra (DARTEL) technique (Ashburner 2007). Finally, each filtered functional volume was spatially normalized to MNI space using the deformation parameters estimated during the above step and resampled into a 3-mm cubic voxel. After spatial normalization, all data sets were smoothed with a Gaussian kernel of 6 × 6 × 6 mm^3^ full-width at half maximum (FWHM).

**References**

**Supplementary figures and tables**


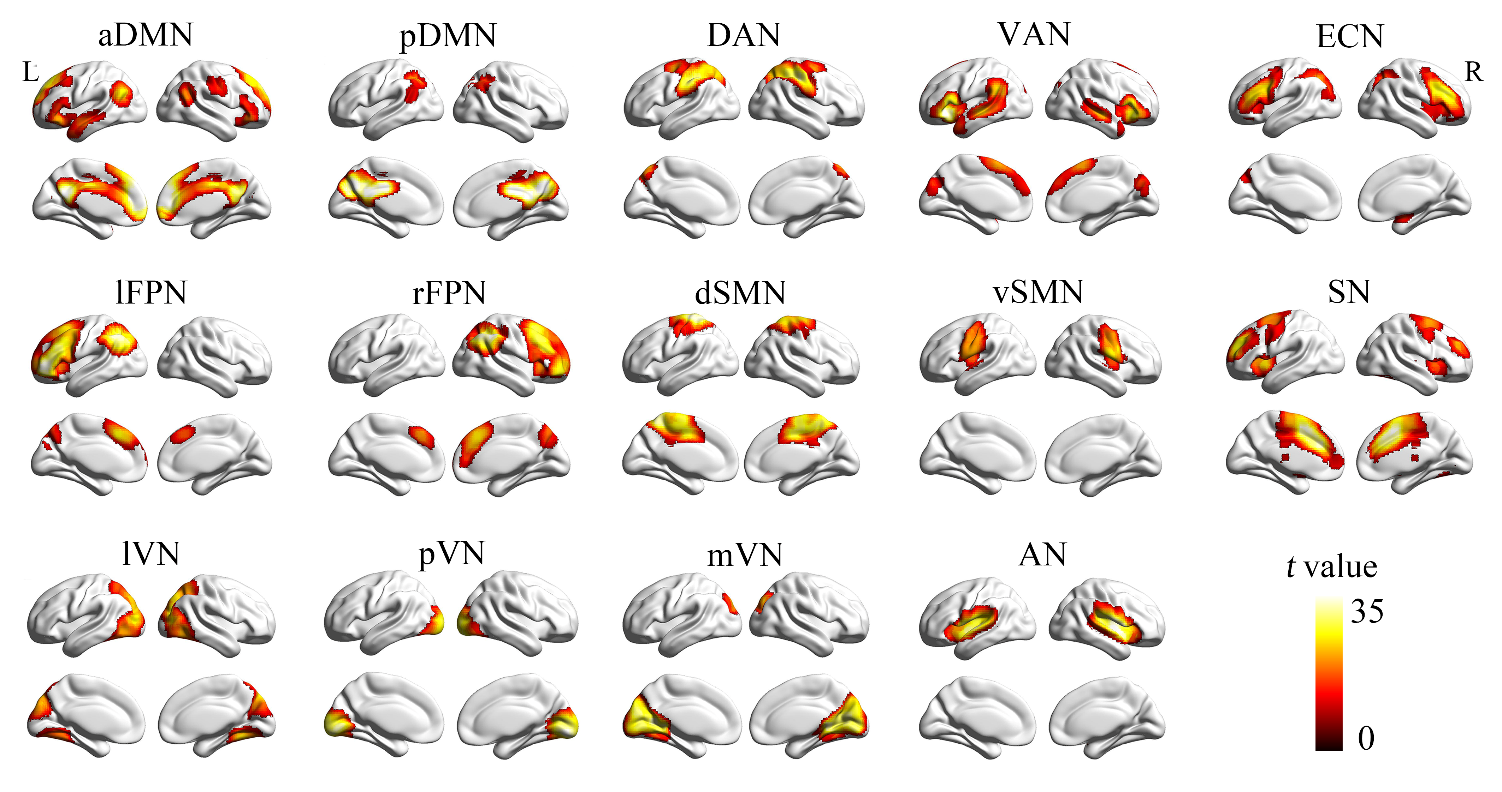


**Figure S1.** Spatial maps of 14 selected functional networks. The color scale represents *t* value. Abbreviations: aDMN, anterior default mode network; AN, auditory network; DAN, dorsal attention network; dSMN, dorsal sensorimotor network; ECN, executive control network; L, left; lFPN, left frontoparietal network; lVN, lateral visual network; mVN, medial visual network; pDMN, posterior default mode network; pVN, posterior visual network; R, right; rFPN, right frontoparietal network; SN, salience network; VAN, ventral attention network; vSMN, ventral sensorimotor network.

**Table S1.** Correlations between gut *Bacteroides* and brain-related metabolic pathways

| Bacteria | Metabolic pathways | *pr* | *p* |
| --- | --- | --- | --- |
| *Bacteroides* | Alanine, aspartate and glutamate metabolism | 0.196 | > 0.05 |
| *Bacteroides* | Arginine and proline metabolism | 0.332 | **< 0.001** |
| *Bacteroides* | Biosynthesis of unsaturated fatty acids | -0.530 | **< 0.001** |
| *Bacteroides* | D-Alanine metabolism | -0.805 | **< 0.001** |
| *Bacteroides* | D-Arginine and D-ornithine metabolism | -0.266 | **0.027** |
| *Bacteroides* | D-Glutamine and D-glutamate metabolism | -0.731 | **< 0.001** |
| *Bacteroides* | Fatty acid biosynthesis | -0.399 | **< 0.001** |
| *Bacteroides* | Fatty acid degradation | 0.361 | **< 0.001** |
| *Bacteroides* | Glutathione metabolism | 0.040 | > 0.05 |
| *Bacteroides* | Glycerolipid metabolism | -0.570 | **< 0.001** |
| *Bacteroides* | Glycerophospholipid metabolism | -0.379 | **< 0.001** |
| *Bacteroides* | Glycine, serine and threonine metabolism | -0.339 | **< 0.001** |
| *Bacteroides* | Histidine metabolism | -0.153 | > 0.05 |
| *Bacteroides* | Insulin signaling pathway | -0.396 | **< 0.001** |
| *Bacteroides* | Linoleic acid metabolism | 0.014 | > 0.05 |
| *Bacteroides* | Lipopolysaccharide biosynthesis | 0.133 | > 0.05 |
| *Bacteroides* | Lysine biosynthesis | -0.610 | **< 0.001** |
| *Bacteroides* | Lysine degradation | -0.159 | > 0.05 |
| *Bacteroides* | Phenylalanine metabolism | 0.546 | **< 0.001** |
| *Bacteroides* | Phenylalanine, tyrosine and tryptophan biosynthesis | -0.107 | > 0.05 |
| *Bacteroides* | Primary bile acid biosynthesis | 0.578 | **< 0.001** |
| *Bacteroides* | Pyruvate metabolism | 0.130 | > 0.05 |
| *Bacteroides* | Riboflavin metabolism | -0.545 | **< 0.001** |
| *Bacteroides* | Secondary bile acid biosynthesis | 0.578 | **< 0.001** |
| *Bacteroides* | Sphingolipid metabolism | 0.689 | **< 0.001** |
| *Bacteroides* | Starch and sucrose metabolism | -0.197 | > 0.05 |
| *Bacteroides* | Steroid hormone biosynthesis | 0.926 | **< 0.001** |
| *Bacteroides* | Synthesis and degradation of ketone bodies | -0.511 | **< 0.001** |
| *Bacteroides* | Tryptophan metabolism | -0.221 | > 0.05 |
| *Bacteroides* | Tyrosine metabolism | 0.170 | > 0.05 |
| *Bacteroides* | Valine, leucine and isoleucine biosynthesis | -0.494 | **< 0.001** |
| *Bacteroides* | Vitamin B6 metabolism | -0.059 | > 0.05 |
| *Bacteroides* | beta-Alanine metabolism | -0.112 | > 0.05 |

*p* values were corrected for multiple comparisons using the Bonferroni method. Bold *p* values are statistically significant. Abbreviations: *pr*, partial correlation coefficient.
